# Malaria Parasitemia among Febrile Patients Seeking Clinical Care at an Outpatient Health Facility in an Urban Informal Settlement Area in Nairobi, Kenya

**DOI:** 10.4269/ajtmh.15-0293

**Published:** 2016-01-06

**Authors:** Henry N. Njuguna, Joel M. Montgomery, Leonard Cosmas, Newton Wamola, Joseph O. Oundo, Meghna Desai, Ann M. Buff, Robert F. Breiman

**Affiliations:** Kenya Medical Research Institute (KEMRI), Nairobi, Kenya; U.S. Centers for Disease Control and Prevention, Center for Global Health, Kisumu and Nairobi Kenya, and Atlanta, Georgia; U.S. President's Malaria Initiative, Nairobi, Kenya

## Abstract

Nairobi is considered a low-risk area for malaria transmission, but travel can influence transmission of malaria. We investigated the demographic characteristics and travel history of patients with documented fever and malaria in a study clinic in a population-based surveillance system over a 5-year period, January 1, 2007 to December 31, 2011. During the study period, 11,480 (68%) febrile patients had a microscopy test performed for malaria, of which 2,553 (22%) were positive. Malaria was detected year-round with peaks in January, May, and September. Children aged 5–14 years had the highest proportion (28%) of positive results followed by children aged 1–4 years (23%). Almost two-thirds of patients with malaria reported traveling outside Nairobi; 79% of these traveled to three counties in western Kenya. History of recent travel (i.e., in past month) was associated with malaria parasitemia (odds ratio: 10.0, 95% confidence interval: 9.0–11.0). Malaria parasitemia was frequently observed among febrile patients at a health facility in the urban slum of Kibera, Nairobi. The majority of patients had traveled to western Kenya. However, 34% reported no travel history, which raises the possibility of local malaria transmission in this densely populated, urban setting. These findings have important implications for malaria control in large Nairobi settlements.

## Introduction

Since 2000, the burden of malaria in Africa has significantly declined^1^; the decline has been attributed to substantial coverage increases in four main malaria prevention and control activities: the distribution of long-lasting insecticide-treated bed nets (LLINs), indoor residual spraying of insecticides, intermittent preventive treatment of pregnant women, and effective case management.^2^,^3^ However, malaria remains a leading cause of morbidity and mortality in Kenya where approximately 21% of all outpatient visits are for malaria-related illnesses.^4^

Kenya has four eco-epidemiological malaria zones: endemic Lake Victoria and coastal counties; epidemic-prone highland counties; seasonal transmission counties; and counties with no-to-very-low transmission risk, including Nairobi County, primarily due to high altitude and low seasonal temperatures.^4^,^5^ Among children < 15 years of age, malaria parasite prevalence varies across the eco-epidemiological zones from a high of 38% in the lake-endemic counties to a low of ≤ 1% in low-risk counties.^5^ Efforts to reduce the burden of malaria through the four main prevention and control strategies have focused primarily on areas with high malaria prevalence.^4^,^6^

Travel and migration of people between regions can influence malaria transmission^7^ and contributes to the geographic spread of resistance to malaria medications.^8^ Nairobi, like other large cities in developing countries, is experiencing massive and rapid urbanization. Rural populations migrate in search of economic opportunity, which has resulted in more people living in crowded, informal settlements (i.e., slums). An estimated 1.9 million people or 60% of the total population of Nairobi lives in informal settlements.^9^ Unlike many developing countries, Kenya has a relatively well-developed transportation infrastructure that facilitates frequent travel of workers and families from Nairobi to their rural homes in western Kenya and the coastal region, where malaria is holoendemic. Travelers become infected with malaria while visiting endemic areas and are parasitemic on return to Nairobi.^10^ If *Anopheles* mosquito vectors are present, as documented by Okara and others, and environmental factors are conducive, *Plasmodium* species could be introduced with resultant local transmission.^11^

Understanding the characteristics and movement patterns of people infected with malaria parasites is important for successful and targeted intervention strategies to control malaria transmission.^7^,^12^–^14^ We describe the demographic features of febrile patients with malaria parasitemia, identify the geographical regions to which they frequently traveled, and assess the seasonal patterns of malaria infection among patients seeking care at an outpatient clinic in the Kibera slum in Nairobi, Kenya, from 2007 to 2011.

## Methods

### Study area.

Since 2005, the Kenya Medical Research Institute (KEMRI) and the U.S. Centers for Disease Control and Prevention (CDC) have collaborated on a population-based infectious disease surveillance (PBIDS) project within two villages of Kibera, an urban, informal settlement in Nairobi.^15^ Kibera is located 5 km southwest of the central business district of Nairobi. Nairobi's altitude is above 1,530 m, and Kibera has a fairly uniform altitude that ranges from 1,719 to 1,748 m.^16^,^17^ An estimated 70% of residents of the surveillance area migrated from western Kenya, where malaria is holoendemic.^18^,^19^ The majority of Kibera residents regularly travel to their rural homes for temporary visits (e.g., for seasonal farm work and holidays).

### Surveillance procedures.

The Kibera PBIDS focuses on the burden and etiologies of febrile illness, diarrheal disease, pneumonia, and jaundice within two of 13 villages in Kibera: Gatwikera and West Soweto.^15^ The surveillance system has been previously described.^15^,^20^ Eligible persons must have resided permanently in these two villages for 4 calendar months or be a child born to a woman enrolled in PBIDS. Household visits were conducted by community interviewers every 2 weeks to enroll new participants and inquire about illnesses during the past 2 weeks. Enrollment was continuous after surveillance commencement. Participants who were not documented to be residing within the household for eight consecutive biweekly visits (4 calendar months) were excluded from the surveillance. During the study period, the average population of the Kibera surveillance area was approximately 30,700 (range = 27,619–34,503) within a 0.37 km^2^ area (population density = 83,000 persons/km^2^).

Surveillance participants with acute medical illnesses had free access to a study clinic with trained staff, laboratory testing, and medications. The clinic is within 1 km of all residences within the surveillance area. During clinic visits, clinical and demographic data were systematically collected using structured questionnaires. In addition, patients were asked whether they had traveled outside Nairobi during the previous 1 month. Data were entered by project staff directly into a computerized, standardized database. Patients with measured fever (i.e., axillary temperature ≥ 37.5°C) and symptoms or signs suggestive of malaria (e.g., headache, vomiting, and muscle and joint pains) had blood collected for malaria microscopy. Patients who tested positive for malaria were treated following Kenya national malaria treatment guidelines with artemether–lumefantrine, the recommended first-line treatment of uncomplicated malaria.^21^

### Laboratory procedures.

Thick blood smears were prepared and stained with 3% Giemsa for 10 minutes following World Health Organization standards.^22^,^23^ The slides were viewed at 100× power magnification under oil immersion. A slide was considered positive if a single asexual malaria parasite was seen by a qualified laboratory technologist after examining at least 200 high-power fields. Parasite densities were not computed for this study.

### Data analysis.

We analyzed data using STATA (version 9.2; Stata Corporation, College Station, TX). In the analysis, we considered only records of patients with measured fever (temperature ≥ 37.5°C) who were tested for malaria. A malaria case was defined as a patient with a measured fever (temperature ≥ 37.5°C) and a positive blood smear test result. For patients who had repeat visits within a period of < 2 weeks and had documented fever plus positive malaria blood slides in both the initial and repeat visits, we only included the initial malaria diagnosis in the analysis.

We determined the demographic characteristics of febrile patients and malaria cases visiting the study clinic and identified factors associated with having a positive malaria test result. To compare risk by age, we used the following age groupings: < 6 months (referent), 6–11 months, 1–4 years, 5–14 years, and ≥ 15 years.

For patients reporting travel in the last month prior to the clinic visit, we identified the counties they visited and described the characteristics of those who had traveled. For each county visited, we determined the number of travelers that had malaria parasitemia and compared these numbers to transmission rates in the Kenya Ministry of Health's District Health Information System 2 (DHIS2) (Ministry of Health, unpublished data).

For patients without travel history, we described the age and sex distribution and identified months with the highest proportion of malaria cases. Finally, we described the monthly distribution of all malaria cases over the 5-year study period and stratified them based on travel status.

### Ethical review.

The protocol, surveillance questionnaires, and consent forms were reviewed and approved by the Ethical Review Committee at KEMRI (protocol 1899) and the Institutional Review Board at CDC (protocol 4566).

## Results

During the 5-year study period, 105,960 patient visits occurred at the study clinic (Table 1). Of all patient visits, preschool children aged 1–4 years (*N* = 36,630, 35%) and adults ≥ 15 years (*N* = 36,059, 34%) had the highest number of clinic visits. Females accounted for 56% of all visits. Of all patients, 16% had a measured temperature of ≥ 37.5°C on presentation; infants, aged 6–11 months, were responsible for the highest proportion (21%) followed by children aged 1–4 years (20%) and 5–14 years (18%) (Table 1).

Among febrile patients, 68% (*N* = 11,480) had a blood slide processed for malaria microscopy. The range was 42% in infants aged < 6 months to 72% in children aged 5–14 years. Compared with infants < 6 months of age, the likelihood of being tested for malaria increased with age up to a peak in children aged 5–14 years. Patients with history of travel outside Nairobi within a month prior to clinic visit were over 6.6 times more likely to be tested for malaria compared with patients who did not travel (95% confidence interval [CI] = 5.8–7.6) (Table 2).

Overall, among febrile patients with malaria test results, asexual malaria parasites were seen in 22% (*N* = 2,553) (Table 3). Prevalence of parasitemia ranged from a low of 13% in infants (aged < 6 months) to a peak of 28% in children aged 5–14 years (Table 3).

Compared with infants aged < 6 months, febrile children in the age categories 1–4 years, 5–14 years, and adults ≥ 15 years had increased odds of having malaria parasites (Table 3). There was no significant difference in the distribution of malaria parasitemia by gender (*P* = 0.1). Febrile patients who reported having traveled outside Nairobi during the month prior to clinic visit were 10 times more likely to have malaria parasitemia (95% CI = 9.0–11.0) (Table 3).

**Figure 1.**
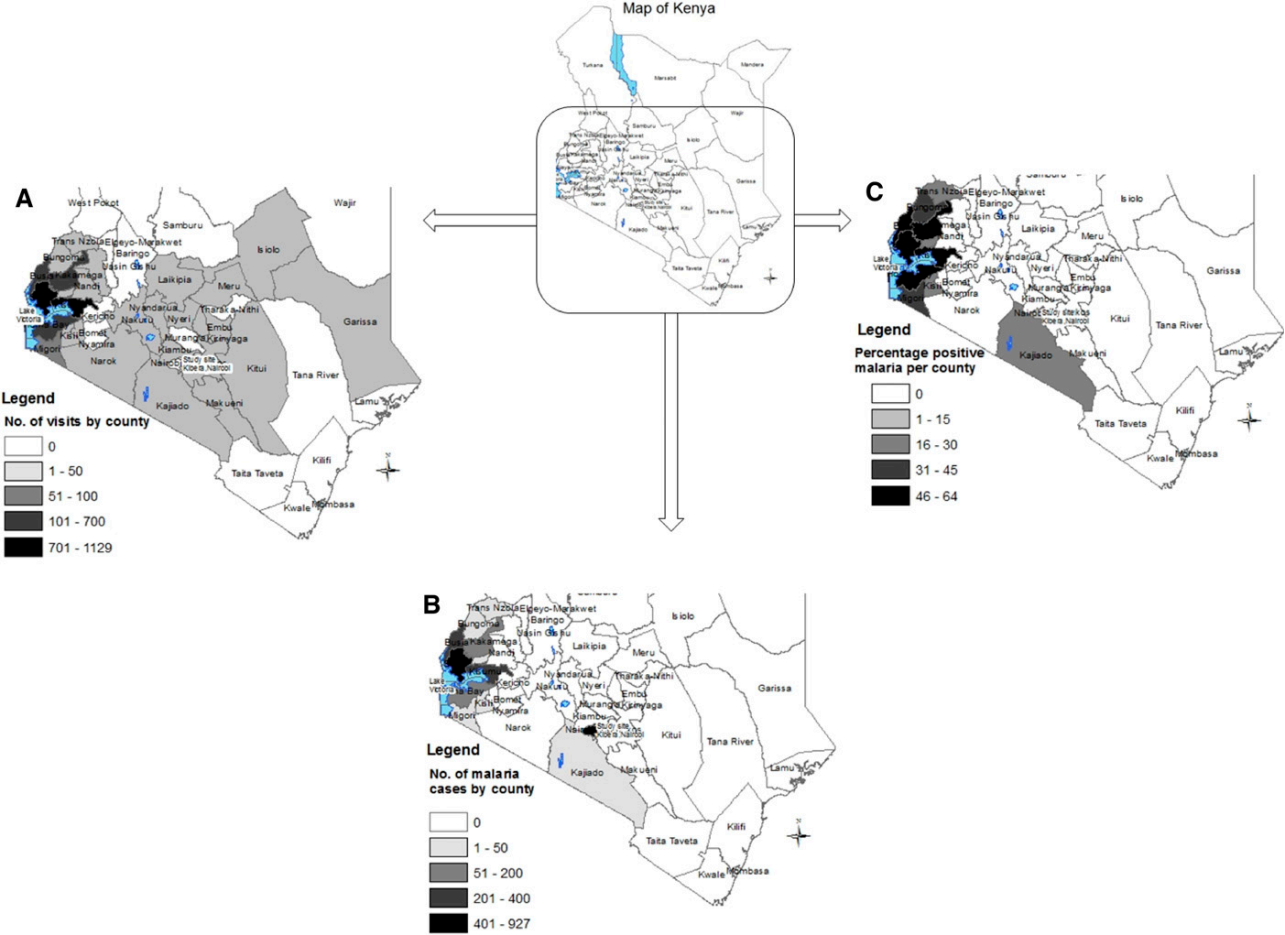
(**A**) Number of visits, (**B**) distribution of malaria cases, and (**C**) percent positive malaria cases by county among patients seen in Kibera clinic, Nairobi, Kenya, from January 1, 2007 to December 31, 2011.

**Figure 2.**
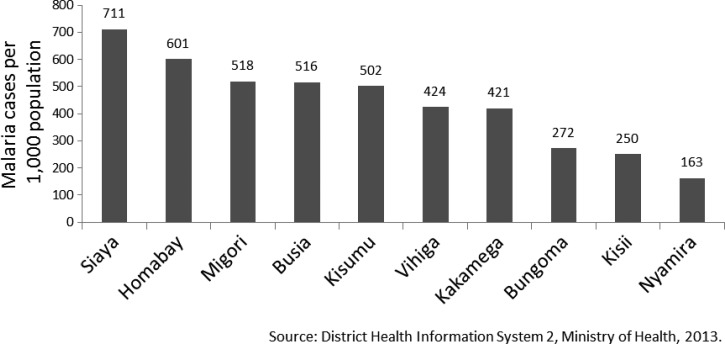
Total reported malaria cases per 1,000 populations in selected counties of western Kenya, 2013.

Of malaria cases, 64% reported traveling outside Nairobi in the month prior to diagnosis; 79% (1,275/1,623) of whom reported travel to just three counties: Siaya (44%), Kisumu (21%), and Busia (13%) (Figure 1

). These three counties represent three of the five counties with the highest malaria cases per 1,000 persons in 2013 from routine health data reported in Kenya's DHIS2 (Figure 2

).

**Figure 3.**
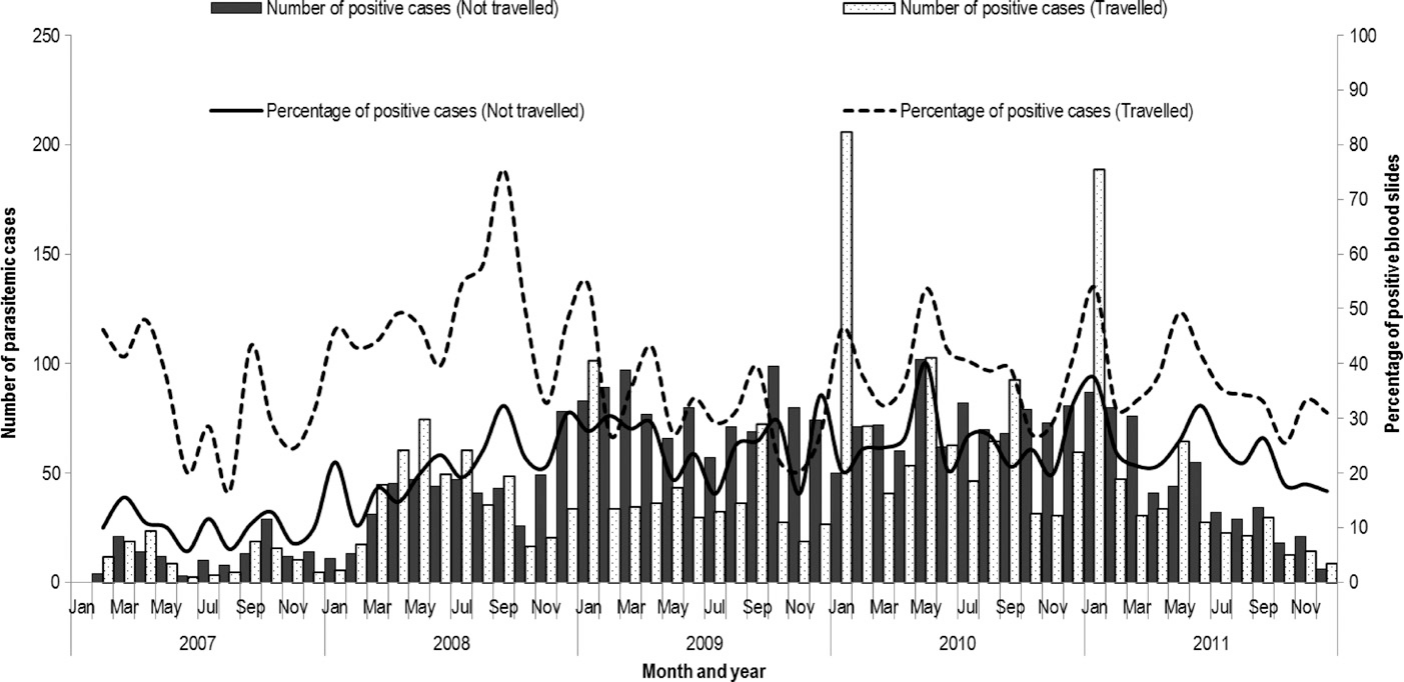
Number of malaria cases and percentage of blood smears positive by travel status in the study clinic, Kibera, Nairobi, Kenya, from January 1, 2007 to December 31, 2011.

Febrile patients with malaria parasitemia were seen year-round with peaks in the months of January, April–May, and August–September, coinciding with end of school holidays (Figure 3

).

## Discussion

This study describes the epidemiology of malaria cases evaluated at an outpatient clinic in Kibera slum in Nairobi. Although Nairobi is considered a low-risk area for malaria transmission due to high altitude and low temperatures, over 20% of febrile residents visiting the study clinic had malaria parasitemia over the 5-year study period and children < 15 years of age accounted for two-thirds of malaria cases. Previous studies in Nairobi have estimated wide ranges of malaria prevalence.^16^,^19^ A study that enrolled asymptomatic community children aged 6 months to 14 years in Kibera to evaluate soil-transmitted helminth infections and nutritional status found an overall malaria parasitemia prevalence of 6.5%.^19^ Routine national health data combined with audits of 14 randomly selected health facilities with laboratory capacity within Nairobi demonstrated a mean blood slide positivity of 15% (range = 4–31%), regardless of patients' clinical presentation.^16^ Therefore, our finding of 22% malaria prevalence among febrile patients in Kibera is consistent with previous studies.

Two-thirds of patients with malaria in our study reported history of travel outside Nairobi within a month prior to diagnosis. Just three (7%) of 46 counties accounted for more than three-quarters of all destinations visited by patients with malaria. These three counties; Siaya, Kisumu, and Busia, which border Lake Victoria, also have three of the five highest rates of malaria in Kenya. Our study confirms findings by Wesolowski and others that described the primary malaria parasite “source” as centered in western Kenya, including parts of Siaya and Busia counties, and the primary “sink” centered in Nairobi using malaria prevalence and mobile phone data to analyze the travel patterns of nearly 15 million individuals.^10^ In addition, malaria prevalence tended to increase during periods that coincided with the end of school holidays when families and children travel back to Nairobi after visiting their rural homes primarily in western Kenya. Our study suggests that communicating risk to travelers and encouraging preventive measures, such as consistent use of LLINs when visiting western Kenya, should be more widely communicated in focused urban areas. Incorporating effective malaria prevention messaging into education programs in schools could reach those most affected, that is, school-aged children, and is consistent with the National Malaria Strategy 2009–2017.^6^ Conversely, implementing malaria prevention and control strategies effectively in holoendemic counties, that is, malaria parasite source counties, might result in a decrease in the number of malaria cases imported to Nairobi and other urban centers in Kenya.

Our study also raises the possibility of local transmission of *Plasmodium* in the densely populated urban Kibera slum. Approaches that could help resolve the question of local transmission in Kibera include entomologic studies to determine the presence and density of *Anopheles* species in Kibera and mapping and genetic characterization of *Plasmodium* species in Kibera and western Kenya to assess movement of parasites with similar genomic patterns between the two areas. Vector data collected from 2001 to 2003 in Kibera demonstrated evidence of the mosquito vector *Anopheles gambiae* s.l. breeding in polluted water pools.^24^ However, *An. gambiae* s.l. mosquitos represented only 0.05% of the almost 177,000 mosquitos captured and speciated, and none of the mosquitos were found to be positive for *Plasmodium* sporozoites.^24^ Although not evaluated in this study, climatic factors, such as rising surface temperatures, have been implicated in widened *Anopheles* habitats resulting in the potential for increased malaria transmission in highland areas in east Africa.^25^,^26^ However, other studies have disputed the role of climate change as a primary factor contributing to the increased malaria transmission in this region.^27^ The relatively cooler temperatures in Nairobi, with lows of 11–14°C, are thought to limit the development of the *Plasmodium* sporozoite stage in the salivary glands of the mosquito vector.^16^,^28^ If seasonal temperatures were to increase, potential windows of transmission could exist in Nairobi in the presence of appropriate vectors.^16^ Because of massive urban migration in Nairobi over the last decade largely from holoendemic counties in western Kenya and concomitant changes in temperature and rainfall patterns in eastern Africa, including increases in minimum temperatures,^29^ urban slums in Nairobi might now have the vectors, environmental conditions, and a sufficient number of parasitemic residents to initiate local *Plasmodium* transmission.

One limitation of the study was the time frame associated with the question elucidating travel history. Over one-third of patients with malaria reported they had not traveled in the previous 1 month prior to presentation. The 1-month window used to define travel history was likely insufficient to differentiate between malaria acquired during travel and malaria potentially acquired in Nairobi. Malaria parasitemia can persist for prolonged periods of up to a year with mild or no symptoms in persons with partial or full immunity living in endemic areas.^30^ Patients who had not traveled but had malaria parasitemia might have recently migrated to Nairobi from a malaria-endemic county or have been exposed to malaria during earlier travel episodes, which would not have been captured. This limitation could have been minimized by asking about travel history over a longer period prior to illness. Second, patients with acquired partial immunity to malaria, especially older children and adults, might have fever and symptoms due to another illness and not malaria. Detection of malaria parasites in patients with acquired immunity would have been an incidental finding, which would have led to an overestimation of malaria cases among patients. Malaria as an incidental finding would have differentially affected patients with a travel history because those patients were more likely to be tested for malaria. Third, we relied on microscopy to detect malaria parasites. Identification of *Plasmodium* by light microscopy relies on the skills of the laboratory technician. Despite having well-trained, experienced microscopists, the potential for human error would lead to misclassification of cases. Another limitation of the study was the potential for recall or information bias. Patients, particularly children or caregivers, might not have remembered or reported travel in the past month or not considered returning to a rural home as travel. However, underreporting of travel by patients, if addressed, would likely strengthen our finding that the majority of malaria infections in Kibera are acquired during travel to malaria endemic areas. Finally, our study was conducted in one slum area within Nairobi. The majority of the slum residents studied had their rural homes in western Kenya where they frequently traveled. Findings from this study are therefore not generalizable to the greater Nairobi slum settings whose residents might not be from primarily malaria-endemic regions.

Our study demonstrates that malaria parasitemia is relatively common in febrile patients in Kibera, Nairobi, both with and without a recent history of travel. Although the findings do not confirm local transmission of malaria, further focused entomologic investigations, improved surveillance for malaria, enhanced diagnostic capacity to confirm parasitemia, and implementation of an effective communications strategy to prevent and control malaria in urban informal settlements are needed.^31^

## Conclusion

The majority of malaria cases reported having traveled to three counties in western Kenya that have the highest rates of malaria in the country. Eliminating malaria in these counties, as well as communicating and implementing effective malaria prevention strategies targeted at travelers to counties in western Kenya, is likely to reduce the malaria burden in Kibera, Nairobi.

## Figures and Tables

**Table 1:** Age and sex characteristics of patients visiting study clinic between January 1, 2007 and December 31, 2011, Kibera, Nairobi, Kenya

Characteristics	All clinic visits		Patients with measured fever (≥ 37.5°C)		Patients with malaria microscopy test	
	*N*	%	*N*	%	*N*	%
Age category						
< 6 months	5,485	5	790	14	331	42
6–11 months	7,651	7	1,593	21	954	60
1–4 years	36,630	35	7,490	20	5,137	69
5–14 years	20,135	19	3,814	18	2,735	72
≥ 15 years	36,059	34	3,286	9	2,323	71
Total	105,960	100	16,973	16	11,480	68
Sex						
Males	47,104	44	8,314	18	5,677	68
Females	58,856	56	8,659	15	5,803	67
Total	105,960	100	16,973	16	11,480	68

**Table 2:** Characteristics associated with malaria testing among febrile patients evaluated at a clinic in Kibera, Nairobi, Kenya, from January 1, 2007 to December 31, 2011

Characteristics	Tested for malaria		Bivariate analysis		
	No	Yes		
	*n* (%)	*n* (%)	OR	*P* value	95% CI
Age category					
< 6 months	459 (58)	331 (42)	Ref	–	–
6–11 months	639 (40)	954 (60)	2.1	< 0.01	1.7–2.5
1–4 years	2,353 (31)	5,137 (69)	3.0	< 0.02	2.6–3.5
5–14 years	1,079 (28)	2,735 (72)	3.5	< 0.03	3.0–4.1
≥ 15 years	963 (29)	2,323 (71)	3.4	< 0.04	2.9–3.9
Total	5,493 (32)	11,480 (68)			
Sex					
Male	2,637 (32)	5,677 (68)	Ref	–	–
Female	2,856 (33)	5,803 (67)	1.0	0.08	0.9–1.0
Travel status					
No	4,434 (35)	8,072 (65)	Ref	–	–
Yes	249 (8)	2,991 (92)	6.6	< 0.01	5.8–7.6
Unknown	883 (68)	417 (32)	0.3	< 0.01	0.3–0.3

CI = confidence interval; OR = odds ratio; Ref = reference.

**Table 3:** Demographic predictors of malaria parasitemia among febrile patients evaluated at a clinic in Kibera, Nairobi, Kenya, from January 1, 2007 to December 31, 2011

Characteristics	Malaria test results		Bivariate analysis		
	Negative	Positive		
	*n* (%)	*n* (%)	OR	*P* value	95% CI
Age category					
< 6 months	287 (87)	44 (13)	Ref	–	–
6–11 months	824 (86)	130 (14)	1.0	0.88	0.7–1.5
1–4 years	3,966 (77)	1,171 (23)	1.9	< 0.01	1.4–2.7
5–14 years	1,958 (72)	777 (28)	2.6	< 0.01	1.9–3.6
≥ 15 years	1,892 (81)	431 (19)	1.5	0.02	1.1–2.1
Total	8,927 (78)	2,553 (22)			
Sex					
Male	4,378 (77)	1,229 (23)	Ref	–	–
Female	4,549 (78)	1,254 (22)	0.9	0.10	0.9–1.0
Travel status					
No	7,214 (89)	858 (11)	Ref	–	–
Yes	1,368 (46)	1,623 (54)	10.0	< 0.01	9.0–11.0
Unknown	345 (83)	72 (17)	1.8	< 0.01	1.3–2.3

CI = confidence interval; OR = odds ratio; Ref = reference.
